# Bioprospecting potential of halogenases from Arctic marine actinomycetes

**DOI:** 10.1186/s12866-016-0662-2

**Published:** 2016-03-10

**Authors:** Li Liao, Ruiqin Chen, Ming Jiang, Xiaoqing Tian, Huan Liu, Yong Yu, Chenqi Fan, Bo Chen

**Affiliations:** SOA Key Laboratory for Polar Science, Polar Research Institute of China, 451 Jinqiao Road, Shanghai, 200136 China; College of Bioengineering, East China University of Science and Technology, Shanghai, 200237 China; State Key Laboratory of Microbial Metabolism, School of Life Sciences and Biotechnology, Shanghai Jiao Tong University, Shanghai, 20030 China; Key Laboratory of East China Sea & Oceanic Fishery Resources Exploitation and Utilization, Ministry of Agriculture, East China Sea Fisheries Research Institute, Chinese Academy of Fishery Sciences, Shanghai, 200090 China; College of Marine Sciences, Shanghai Ocean University, Shanghai, 201306 China

**Keywords:** Halogenase, Actinomycetes, Arctic, Bioprospecting

## Abstract

**Background:**

Halometabolites, an important group of natural products, generally require halogenases for their biosynthesis. Actinomycetes from the Arctic Ocean have rarely been investigated for halogenases and their gene clusters associated, albeit great potential of halometabolite production has been predicted. Therefore, we initiated this research on the screening of halogenases from Arctic marine actinomycetes isolates to explore their genetic potential of halometabolite biosynthesis.

**Results:**

Nine halogenase genes were discovered from sixty Arctic marine actinomycetes using in-house designed or previously reported PCR primers. Four representative genotypes were further cloned to obtain full coding regions through genome walking. The resulting halogenases were predicted to be involved in halogenation of indole groups, antitumor agent ansamitocin-like substrates, or unknown peptide-like compounds. Genome sequencing revealed a potential gene cluster containing the halogenase predicted to catalyze peptide-like compounds. However, the gene cluster was probably silent under the current conditions.

**Conclusions:**

PCR-based screening of halogenase genes is a powerful and efficient tool to conduct bioprospecting of halometabolite-producing actinomycetes from the Arctic. Genome sequencing can also identify cryptic gene clusters potentially producing new halometabolites, which might be easily missed by traditional isolation and chemical characterization. In addition, our study indicates that great genetic potential of new halometabolites can be expected from mostly untapped actinomycetes from the polar regions.

**Electronic supplementary material:**

The online version of this article (doi:10.1186/s12866-016-0662-2) contains supplementary material, which is available to authorized users.

## Background

Actinomycetes are especially important in producing various bioactive secondary metabolites, including well-known antibiotics such as chloramphenicol [1] and anticancer molecules such as rebeccamycin [2]. Actinomycetes isolated from terrestrial environments have been well studied, which results in frequent rediscovery of known natural products [3, 4]. In contrast, marine actinomycetes from the deep ocean or the polar regions are mostly untapped resources [5]. Currently, new actinomycetes have been described from the Arctic and Antarctica, using cultivation-dependent and -independent approaches [6–9]. An increasing number of natural products have been reported from marine actinomycetes as well [5, 10].

To meet the urgent medical needs of drug candidates, advanced approaches are required to improve the efficiency of natural product discovery [11–13]. Generally, similar secondary metabolites are biosynthesized by gene clusters that contain certain homologous genes [14, 15]. The presence of marker genes, such as polyketide synthase (PKS), non-ribosomal peptide synthetase (NRPS) genes and tailoring enzyme genes, may indicate production of certain secondary metabolites. Therefore, PCR-based marker gene screening is an efficient way to find promising natural products from the environment [16].

Halogenation catalyzed by halogenases is an important tailoring step for bioactivities of many natural products [17, 18]. Halogenated natural products span from simple halogenated indoles, terpenes and phenols to complex oligopeptides and polyketides. Halogenated compounds are important sources for new drugs, due to their high diversity in structure and activity. More than 4,700 halogenated compounds have been reported, showing diverse biological activities and structures [19]. Some of them have been used for decades as pharmaceuticals. For example, the well-known antibiotic chloramphenicol [1], the antitumor agent rebeccamycin [2] and the antifugal antibiotic pyrrolnitrin [20] have been used widely in clinic or currently under clinical trials. Therefore, exploration of halometabolites is an important and promising approach to discover new drugs. In addition to potential biotechnological applications, antibiotics including halometabolites are considered to be bacterial weapons for fighting competitors in the indigenous ecosystems or signals regulating the homeostasis of the microbial communities [21]. Therefore, they may also have important ecological functions. Halogenase is a key tailoring enzyme in producing halometabolites in nature and can be used to explore halometabolites. Currently, halogenases can be divided into two types [22]. One type is highly substrate-specific, including flavin adenine dinucleotide (FADH_2_)-dependent halogenases (FDHs), non-heme Fe^II^/α-ketoglutarate halogenases and SAM-dependent halogenases. The other type is haloperoxidases that generally lack of substrate specificity. FDHs are the major type of halogenases involved in biosynthesis of halometabolites, and are usually indicative of halometabolite types [23–25]. One group of well-studied FDHs is tryptophan halogenases (Trp-halogenases) that regioselectively halogenate tryptophan (e.g., [26–30]).

Halogenases from the polar regions and deep sea have been much less investigated than from terrestrial environments and coasts. PCR-based screening of FDHs has been conducted to study marine sponge-associated microbial consortia [31] and actinomycetes from various terrestrial and other environments [32]. FADH_2_-dependent halogenases have been studied in detail from marine and soil actinomycetes, such as *Amycolatopsis* [33] and *Streptomyces* [34], in the context of related gene clusters. However, FDHs from the polar regions are poorly investigated. The Arctic attracts increasing attention due to its unique environmental conditions and strategic importance. Bioprospecting has been carried out in the Arctic for microorganisms in biotechnology [35, 36]. However, few reports have been involved in halogenases. Previously, we detected halogenase genes from Arctic actinomycetes without gene cloning and sequencing [9]. In this study, we screened FDHs from actinomycetes isolated from marine sediments of the high Arctic, and sequenced them for the first time. Novel FDH genes were further cloned to obtain the full coding sequences for analysis. In addition, genome sequencing was performed to discover a cryptic gene cluster predicted to produce an unknown halometabolite. Our study showed that halogenase genes are helpful in bioprospecting Arctic actinomycetes. Great genetic potential of halometabolite production can be expected from the Arctic actinomycetes.

## Results and Discussion

### Bioprospecting for halogenase-containing actinomycetes

**Fig. 1:**
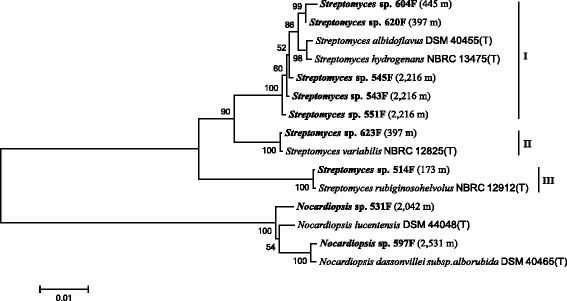
Phylogenetic tree of 16S rRNA genes of halogenase-positive strains and their closest type strains

PCR-based screening was very efficient for bioprospecting of halogenase-containing actinomycetes. Nine out of 60 strains were discovered to contain halogenase genes. The 60 investigated actinomycetes strains were mostly *Streptomyces*, with a few exceptions belonging to *Nocardiopsis*, *Pseudonocardia* and *Brevibacterium* (Additional file 1: Table S1). Moderate frequency of occurrence of halogenases was observed in Arctic actinomycetes, compared with actinomycetes from other environments [16, 32, 37]. The nine strains were distributed at various water depths in the Arctic Ocean (Fig. 1), ranging from shallow (e.g., 514 F at 173 m) to deep (e.g., 597 F at 2,531 m) marine sediments. They grew well at 15 °C in ISPII medium (0.4 % yeast extract, 1.0 % malt extract, 0.4 % glucose), with the optimum temperature around 20 to 25 °C. No correlation between phylogenetic distances and geographic distribution was observed (Fig. 1). The nine halogenase-containing strains were grouped into two genera, *Streptomyces* (seven strains) and *Nocardiopsis* (two strains) (Fig. 1). It agreed with previous studies that *Streptomyces* was the major genus containing halogenases and also one of the most important producers of halometabolites [16, 32, 38]. *Nocardiopsis* is less commonly found containing halogenases. Indeed, no halometabolites have been reported from *Nocardiopsis* to the best of our knowledge. However, higher occurrence of halogenases was observed in *Nocardiopsis* (2 out of 10) than in *Streptomyces* (7 out of 49) in our study (Additional file 1: Table S1), even though the sample size was not large enough to show statistical significance. In addition, the two *Nocardiopsis* strains were isolated from deep-sea sediments of the Arctic Ocean. Hence, the detection of two halogenase-containing *Nocardiopsis* strains is of interest and worthy of further investigation.

The closest type cultures of the nine strains are shown in the phylogenetic tree (Fig. 1), sharing 99 to 100 % 16S rRNA gene identities. The seven *Streptomyces* strains could be clustered into three subgroups in the phylogenetic tree based on 16S rRNA genes (Fig. 1). To identify whether halogenase genes are present in the closely related strains, we searched the NCBI databases for genome and halogenase sequences of them. Only one genome of closely related strains was sequenced, i.e., *Nocardiopsis lucentensis* DSM 44048(T). However, no halogenase genes were found in the draft genome of *Nocardiopsis lucentensis* DSM 44048(T) [GenBank: NZ_ANBC00000000.1]. In addition, no halogenase genes were reported from the remaining closely related strains.

### Analysis of putative halogenase partial sequences

**Fig. 2:**
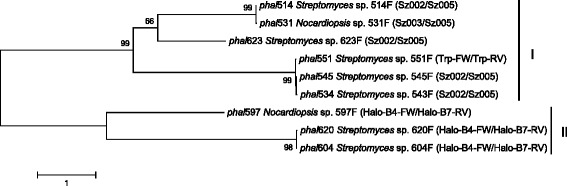
Phylogenetic tree of partial halogenase genes based on DNA sequences

The nine partial putative halogenase genes were amplified by different degenerative primer pairs (Table 1), with five genes amplified by SZ002/SZ003/SZ005, three genes amplified by Halo-B4-FW/Halo-B7-RV, and one gene amplified by Trp-RW/Trp-RV (Fig. 2). It seems that different primer pairs have unequal efficiency, probably related to primer sequences and the conserved domains targeted. Hence, it suggests that more primers used for screening can improve the opportunity of finding halogenases. In addition, false positives occurred more frequently using the primer pairs Halo-B4-FW/Halo-B7-RV and SZ002/SZ003/SZ005 than using the primer pairs Trp-RW/Trp-RV designed in this study. Screening of halogenase genes only based on electrophoresis of PCR products is not reliable. Further cloning and sequencing are required to confirm the presence of halogenase genes.

**Table 1:** Degenerative primers used for screening of halogenase genes

Primer	Sequence (5´- 3´)	Target regions	Reference
Halo-B4-FW	TTCCCSCGSTACCASATCGGSGAG	FPRYHIGES	[16]
Halo-B7-RV	GSGGGATSWMCCAGWACCASCC	GWYWVIPL	
SZ002	TCGGYGTSGGCGARGCGACCRTCCC	GVGEATIP	[26]
SZ003	TSGGCGGCGGCACYGCSGGMTGGATG	GGGTAGWM	
SZ005	GCCGGAGCAGTCGAYGAASAGGTC	DLFIDCSGFR	
Trp-FW	TCGGSGTSGGCGARGCSACCKT	GVGEATF	This study
Trp-RV	CGGTRSWCTCCAGCGGCTCGACGAA	FVEPLESSG

The nine partially sequenced FDHs could be clustered into two groups based on DNA sequences, Trp-halogenases (I) and non-Trp-halogenases (II) (Fig. 2). High homology was shared by halogenase genes from strains with different phylogenetic affiliation. For example, *Streptomyces* sp. 514 F shared almost identical partial halogenase gene (*phal*514) with *Nocardiopsis* sp. 531 F (*phal*531). *Streptomyces* strains 551 F, 545 F and 543 F had nearly identical halogenase partial sequences (*phal*551, *phal*545 and *phal*543). In addition, *Streptomyces* strains 620 F and 604 F contained 99 % identical partial halogenase sequences (*phal*620 and *phal*604). Therefore, halogenases may have complex evolutionary history.

Representative halogenase partial genes were compared against the NCBI GenBank database to search for homologs. The partial halogenase of *Streptomyces* sp. 531 F (pHal531) shared 96 % nucleotide identity and 98 % amino acid identity with Trp-halogenase from *Sphingomonas melonis* [GenBank: WP_017979780]. The closest relative of the partial halogenase from *Streptomyces* sp. 623 F (pHal623) was Trp-halogenases from *Brevundimonas* sp. DS20 (98 % amino acid and nucleotide identities) [GenBank: WP_054765358]. Interestingly, the closest relatives of pHal531 and pHal623 are halogenases from alphaproteobacteria rather than actinomycetes. In addition, these alphaproteobacterial halogenases were either partially cloned or predicted by genome sequencing. Moreover, the alphaproteobacterial halogenases were not located in any potential biosynthetic gene clusters. It raises the question of roles played by halogenases in non-actinomycetes, such as alphaproteobacteria that are not perceived commonly as a natural product-producing group. Whether halogenases in alphaproteobacteria are involved in halometabolite production is still unknown. The closest biochemically characterized halogenases of pHal531 and pHal623 were halogenase RebH from *Lechevalieria aerocolonigenes* ATCC 39243 [GenBank: CAC93722] [30] and halogenase Thal from *Streptomyces albogriseolus* [GenBank: ABK79936] [28], sharing less than 40 % nucleotide and amino acid identities. Hence, pHal531 and pHal623 showed some novelty in sequence and probably in physiological roles as well.

pHal551, representative of pHal545 and pHal543, had highest homology (99 % nucleotide and amino acid identities) with three tryptophan 6-halogenases of *Streptomyces* from mangrove soil. These closest strains are *Streptomyces fungicidicus* strains MGR162 [GenBank: KF425752] and MGR136 [GenBank: KF425746], and *Streptomyces griseus* subsp. *griseus* strain MGR054 [GenBank: KF425729]. However, neither genomes of these mangrove strains were sequenced, nor further studies on their halogenases were published. Therefore, no information of potential gene clusters or products could be obtained from these highly identical halogenases. Nevertheless, pHal551 showed significant identities with halogenases known to be involved in biosynthesis of identified halometabolites. For example, pHal551 had 71 % nucleotide identity and 70 % amino acid identity with Thal from *Streptomyces albogriseolus* [GenBank: ABK79936]. It also showed 75 % nucleotide identity and 70 % amino acid identity with RebH from *Lechevalieria aerocolonigenes* ATCC 39243 [GenBank: CAC93722]. Therefore, pHal551 may resemble the characterized Trp-halogenases, indicating that similar biosynthesis could occur in strain 551 F.

The three non-Trp-halogenases shared high identities with putative halogenases from actinomycetes but low identities with halogenases catalyzing recognized halometabolites. The partial halogenase of *Nocardiopsis* sp. 597 F (pHal597) shared 98 % nucleotide identity and 97 % amino acid identity with putative halogenases from *Streptomyces* sp. NRRL F-6628 [GenBank: WP_037845354] and *Streptomyces griseus* [GenBank: WP_030771994]. However, pHal597 shared only 54 % nucleotide identity and 56 % amino acid identity with the halogenase (Asm12) characterized from *Actinosynnema pretiosum* [39]. pHal620 and pHal604 shared 99 % nucleotide and amino acid identities with halogenase of *Streptomyces* sp. L131(2011) [GenBank: JN035304], but were distinct from halogenases involved in biosynthesis of known halometabolites.

### Analysis and implication of the full-length halogenase genes

**Fig. 3:**
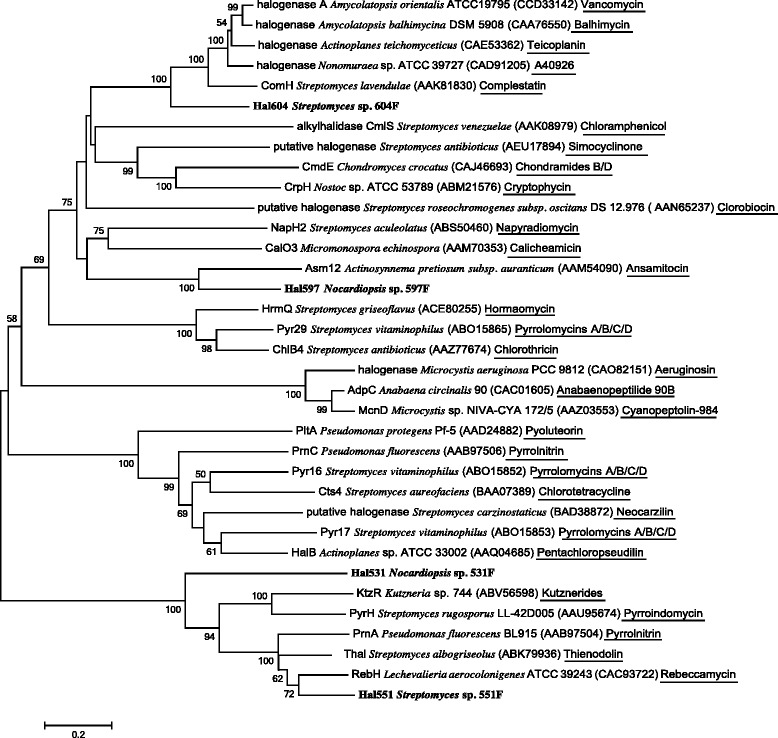
Phylogenetic tree of the full-length halogenases and previous halogenases identified in halometabolite biosynthesis. Phylogenetic tree was built using amino acid sequences. Protein symbols/names, the producing strains, GenBank accession numbers in parentheses, and the corresponding halometabolites underlined were given in order

Since the partial halogenases showed promising novelty and diversity, representatives were chosen to clone full coding sequences. Four full-length halogenase genes were cloned based on genome walking of partial sequences from *Streptomyces* strains 604 F and 551 F, as well as *Nocardiopsis* strains 597 F and 531 F. Both *Nocardiopsis* strains and *Streptomyces* sp. 551 F were isolated from deep-sea sediments from the high Arctic, while *Streptomyces* sp. 604 F was obtained at a shallower depth. Halogenases were reported to usually occur in gene clusters for biosynthesis of related secondary metabolites. Thus, a phylogenetic tree was built for the four complete halogenases and the previously identified halogenases from known biosynthetic gene clusters (Fig. 3).

The complete halogenase gene (*hal*551) of *Streptomyces* sp. 551 F was similar to Trp-halogenases involved in biosynthesis of well-known halometabolites. The *hal*551 gene contained 1,605 bp encoding 534 amino acids. It shared 85 % amino acid identity with multiple putative Trp-halogenases from genome sequencing of *Streptomyces* and 71 % amino acid identity with Thal from *Streptomyces albogriseolus* [GenBank: ABK79936] [28]. Thal was a 6-Trp-halogenase that regioselectively catalyzed chlorination or bromination at the 6-position of tryptophan to produce thienodolin, a growth-regulating factor in plants [28]. The phylogenetic tree showed that Hal551 was phylogenetically related to RebH (67 % amino acid identity) from *Lechevalieria aerocolonigenes* ATCC 39243 [GenBank: CAC93722] [30]. RebH was a Trp-halogenase selectively catalyzing the halogenation of 7-position of tryptophan during the biosynthesis of rebeccamycin. In addition, Hal551 clustered with other known Trp-halogenases, which regioselectively acted on the 5, 6 or 7 position of tryptophan. Therefore, Hal551 is predicted to regioselectively catalyze tryptophan to produce final halometabolites.

Hal531 was probably a new kind of Trp-halogenase. It lay outside of the identified Trp-halogenases and Hal551 in the phylogenetic tree, but was not related to non-Trp-halogenases. The complete gene still had highest identity with halogenases from alphaproteobacteria, but remotely related to actinobacterial halogenases as shown in the tree (Fig. 3). It indicates that Hal531 likely represents an ancestral form of Trp-halogenases that originated from non-actinomycetes such as alphaproteobacteria. However, this hypothesis requires further investigation. Since the function of these closely related halogenases in alphaproteobacteria is unknown, it is of interest to examine the roles of Hal531 in *Nocardiopsis* sp. 531 F.

Hal597 was a non-Trp-halogenase that implied the potential of halometabolite biosynthesis of strain 597 F. The complete gene consisted of 1,203 bp encoding 400 amino acids. Hal597 had highest amino acid identity (98 %) with two putative halogenases in the draft genomes of *Streptomyces* sp. NRRL F-6628 [GenBank: WP_037845354] and *Streptomyces griseus* NRRL F-5618 [GenBank: WP_030771994]. The two *Streptomyces* genomes were highly similar, especially the two contigs (contig 32 of strain NRRL F-6628 and contig 44 of strain NRRL F-5618) containing the halogenases. However, no information on the two *Streptomyces* genomes as well as the halogenases has been published in the literature. In addition to the unidentified putative halogenases, Hal597 shared 54 % amino acid identity with the halogenase (Asm12) from *Actinosynnema pretiosum* subsp. *auranticum* ATCC 31565. Asm12 was identified in the biosynthetic gene cluster of the maytansinoid antitumor agent ansamitocin [39]. The phylogenetic tree also showed that Hal597 clustered with Asm12 with 100 % bootstrap support. Ansamitocin was 19-membered macrocyclic lactams, with chlorine at position C-19 of an aromatic ring. The biosynthetic gene cluster of ansamitocin was split into two parts, which is uncommon. Further investigation of *hal*597 in the context of genome would provide more information on its possible gene cluster and potential products.

Hal604 was predicted to be related to peptide-like halometabolite biosynthesis. The complete gene was 1,443 bp in length encoding 480 amino acids, highly similar with the halogenase gene previously cloned from this strain in our lab [40]. Hal604 shared 99 % amino acid identity with two putative halogenases in genomes of *Streptomyces* sp. GBA 94–10 [GenBank: ESQ01308] and *Streptomyces* sp. PVA 94–07 [GenBank: ESQ07118] isolated from a sponge. The two draft genomes were highly similar and contained almost identical halogenases as well as same flanking genes. The halogenases were located in putative gene clusters which probably produce glycopeptide-like secondary metabolites, as analyzed in antiSMASH [41]. In addition, Hal604 had 58 % amino acid identity with two halogenases of *Actinoplanes* sp. ATCC 53533, which were identified in the biosynthesis of the sulfated glycopeptide UK-68,597 [42]. Moreover, Hal604 clustered with halogenases involved in biosynthesis of vancomycin [33], balhimycin [43], teicoplanin [44], A40926 [45] and complestatin [46], with 100 % bootstrap support in the tree. These products were all built from peptides and modified by halogenation and glycosylation, except complestatin that was a heptapeptide without glycosylation. In addition, halogenation occurred on the benzene rings derived from amino acids of the above peptide-like macrocyclic compounds. Taken together, Hal604 was expected to function in a similar way in the biosynthesis of peptide-like macrocyclic compounds. Since peptide-like macrocyclic natural products are promising in antibacterial activity and other bioactivities, *Streptomyces* sp. 604 F is worthy of further investigation of natural products and the potential gene clusters. However, no halometabolites were detected under the regular conditions using HPLC-TOF MS (High-Performance Liquid Chromatography Time-of-flight Mass Spectrometry) analysis (Additional file 2). In addition, qRT-PCR failed to detect the expression of *hal*604 under the same conditions (Additional file 2). Therefore, the HPLC-TOF MS and qRT-PCR data suggested that *hal*604 was probably silent under the examined conditions.

### Genome mining for target gene cluster

The failure in detection of predicted halometabolites and expression of *hal*604 requires further genomic investigation to detect potential halogenase-containing gene cluster in strain 604 F. Genome was sequenced using Illumina platform. The gap closure and manual correction of the draft genome are still in progress. As expected, a potential gene cluster containing *hal*604 was predicted by using the antiSMASH program. The gene cluster was annotated as in Table 2, containing PKS, modular NRPS genes and genes encoding modification enzymes such as halogenase Hal604. The predicted product of the *hal*604-containing gene cluster was a peptide-derived molecule, corresponding to the prediction based on Hal604 alone. Further investigations of the *hal*604-containing gene cluster are planned to identify the potential halometabolite and its biosynthesis. As an example, it demonstrated that bioprospecting based on halogenase and genomic data mining is promising to discover strains with halometabolite biosynthesis potential. It is especially powerful to discover hidden products usually missed in traditional chemical isolation, since most encoding gene clusters are silent under normal conditions. In addition, halogenases identified in this study could be used in combinatorial biosynthesis to produce new compounds with halogen modification. This was demonstrated previously in PyrH and Thal that halogenate at 5 and 6 positions of tryptophan respectively. Co-expression of PyrH and Thal with rebeccamycin biosynthetic genes produced new derivatives with novel halogenation [47].

**Table 2:** Annotation of the potential halogenase-containing biosynthetic gene cluster

ORF	Annotation
1	cysteine synthase
2	mbtH-like protein
3	halogenase (Hal604)
4	condensation domain-containing protein
5	AMP-dependent synthetase and ligase
6	enoyl-CoA hydratase
7	chalcone and stilbene synthase domain protein (PKS)
8	cytochrome P450
9	phosphopantetheine-binding domain containing protein
10	condensation domain-containing protein (NRPS)
11	cytochrome P450

## Conclusions

New halogenases with indications of potential halometabolite production were discovered from Arctic marine actinomycetes. It showed that halogenase gene-based bioprospecting allowed rapid discovery of microbial strains with potential in halometabolite production. In addition, it could increase the robustness of halometabolite discovery from cryptic or silent gene clusters. Furthermore, it suggested that Arctic marine actinomycetes were promising targets for discovering new halogenases and halometabolites. It encourages further investigation of halometabolite biosynthetic gene clusters and their products from the almost untapped polar microorganisms.

## Methods

### Strains and 16S rRNA gene analysis

A total of 60 actinomycetes strains previously isolated and kept in our lab from marine sediments at various depths (173 m to 2531 m) of the Arctic, sampled during the Third Chinese Arctic Scientific Expedition in 2008, were used for halogenase gene screening (Additional file 1: Table S1). The strains were previously isolated using serial dilution and plating on ISPII agar plates with nalidixic acid (15 μg ml^−1^). The plates were incubated at 15 °C for a few weeks. Colonies were streaked on new agar plates for purification. Purified single colonies were cultured in ISPII liquid medium at 15 °C. Genomic DNA was extracted using the gDNA Bacteria Kit (BioDev-Tech Co., Beijing). Universal bacterial primers 27 F (5´-AGAGTTTGATCCTGGCTCAG-3´) and 1492R (5´-GGTTACCTTGTTACGACTT-3´) were used to amplify 16S rRNA genes. PCR products with the expected size of approximately 1,500 bp were purified using the GENEray Agarose Gel DNA Purification Kit (GENEray Biotechnology Co., Shanghai) and cloned into pMD18-T vectors (TaKaRa). Positive clones of each PCR product were sequenced using 27 F/1492R primers. The resulting 16S rRNA gene sequences were searched against the EzTaxon-e database (http://eztaxon-e.ezbiocloud.net/). Sequences were aligned using the ClustalW algorithm in MEGA 6.0 [48], and phylogenetic trees were constructed accordingly, using the neighbor-joining method with 1,000 bootstrap replicates. Bootstrap values less than 50 were not shown.

### PCR-based screening for putative halogenase genes

Three primer pairs were used to amplify halogenases (Table 1), including two previously designed primer pairs, Halo-B4-FW/Halo-B7-RV [16] and SZ002/SZ003/SZ005 [26], and a new primer pair specific for Trp-halogenases (Trp-FW/Trp-RV) designed in this study. Primers Halo-B4-FW/Halo-B7-RV and SZ002/SZ003/SZ005 targeted FADH_2_-dependent halogenases at different conserved regions (Table 1). Degenerate primers Trp-FW/Trp-RV were designed based on the alignment of known Trp-halogenases PrnA from *Streptomyces viridochromogenes* [GenBank: WP_037891957] and *Streptomyces rimosus* subsp. *rimosus* ATCC 10970 [GenBank: ELQ80637], PyrH from *Streptomyces rugosporus* [GenBank: AFV71318] and *Streptomyces hygroscopicus* subsp. *Jinggangensis* TL101 [GenBank: YP_007697383], and Thal from *Streptomyces albogriseolus* [GenBank: AHD24351] and *Streptomyces toxytricini* [GenBank: ADW94630]. Each 50 μL PCR mixture contained 1 × PCR buffer (without Mg^2+^), 1.5 mM MgCl_2_, 0.2 mM dNTPs, 0.5 μM each primer, 0.2 μg genomic DNA, and 1 U Taq DNA polymerase in final concentration. The touchdown PCR was programmed as follows: 3 min at 95 °C for 1 cycle; 30 s at 95 °C, 45 s at (Tm + 10) °C, 1 min at 72 °C for 10 – 15 cycles; 30 s at 95 °C, 45 s at (Tm or Tm – 5) °C, 1 min at 72 °C for 25 cycles; and 10 min at 72 °C for 1 cycle. PCR products with the expected size of 450 – 900 bp were cloned into pMD18-T vectors (TaKaRa) and sequenced. The resulting sequences were searched against the GenBank database using the BLASTx algorithm. Sequences that did not match to halogenase genes in the database were defined as false positives. DNA sequences of the partial halogenases were aligned with the ClustalW algorithm in MEGA 6.0 to construct a neighbor-joining tree.

### Cloning of full coding sequences of representative halogenases

Two approaches of genome walking were used to amplify the upstream and downstream of partial halogenase genes, including modified thermal asymmetric interlaced (TAIL)-PCR [49, 50], and SiteFinding-PCR [51]. Nested specific primer sets designed for genome walking were listed in Table 3. PCR products with expected size were purified and cloned into pMD18-T vectors for sequencing. The resulting sequences were assembled with the partial halogenase gene sequences to obtain full coding sequences identified by using the NCBI ORF Finder (http://www.ncbi.nlm.nih.gov/gorf/gorf.html). The complete halogenase genes were then analyzed by BLASTn and BLASTp against the GenBank database. Neighbor-joining tree was built for complete halogenases cloned in this study with previous halogenases catalyzing the halogenation of known secondary metabolites.

**Table 3:** Primers designed for genome walking in this study

Gene	Upstream primers (5´- 3´)	Downstream primers (5´- 3´)
531 F	531USP1: GCTCCTTCAGCCAGAATTGATG	531DSP1: GATTACGCCTATCATTTCGATGCCTC
	531USP2: ATCTTGCCGAAGCCGTGGATGT	531DSP2: CGCATCGTCGCCGCCAACCGCC
	531USP3: ATCTTGCCGAAGCCGTGGATGT	531DSP3: ATGGCGACCTGTTCATCGACTGCTC
551 F	551USP1: GTGCTTGCGCTGGAACCAGTAGT	551DSP1: GGCTGGGCGTGGAAGATCCCGATGC
	551USP2: CGAACGGGTGGTAGAAGTGGTCCGGC	551DSP2: GCGAGTTCTGCGAGATGTGGGGGCTGG
	551USP3: CATCTTGAAGCTGGCGTTGCACTCCCGCA	551DSP3: GCCTGGGTGAAGAACGTGGTCAGCATCG
597 F	5GSP597-1: CCTTACGGGCGTGGTCCAGCA	3GSP597-1: GCCCACGTACTGACACAGGCCCAT
	5GSP597-2: CCACCTGCGTCAGTGAACTCG	3GSP597-2: GCCGCCTACGTCGTCGACTCCAGC
	5GSP597-3: CCATGTAGGGCAGCAGAGACTC	3GSP597-3: AGCGGAAAGATCGCCAAGGCATAC
604 F	604USP1: CGCTCACGGACCACGACGCCCTT	604DSP1: GGTGCGCTTCGAGGACGAGAACGG
	604USP2: GAGGAGCATCTGGTCGAACTTCAT 604USP3: TGCCGCCCCGCTTCTTGACGAAC	604DSP2: ACGATGGACTCCAGTCGCCGCTACTCGGACTTCTTCCAG 604DSP3: GACGCGGGCTGGTACTGGTACAT

### Genome sequencing and biosynthetic gene cluster identification

The genome of strain 604 F was sequenced on the Illumina HiSeq 2000 platform at the Beijing Genomics Institute (BGI). Sequences were assembled using SOAPdenovo software [52]. Secondary metabolite biosynthetic gene clusters were predicted by using the Antibiotics and Secondary Metabolites Analysis Shell (antiSMASH) [41].

### Sequence accession numbers

Sequences produced in this study have been submitted to GenBank under the accession numbers below: 16S rRNA gene sequences (KJ017969, KP998446 – KP998453), halogenase gene sequences (KF597545 – KF597550, KP998454 – KP998458), and the biosynthetic gene cluster containing the halogenase gene *hal*604 (KT439326).

## Declarations

### Ethics approval and consent to participate

Not applicable.

### Consent for publication

Not applicable.

### Availability of data and materials

The datasets supporting the conclusions of this article are included within the article and its additional files.

## Additional files

Additional file 1: Table S1. Strains used for halogenase screening. (XLSX 53 kb) Additional file 2: HPLC-TOF MS analysis and qRT-PCR. (PDF 302 kb)

## Abbreviations

PKS: polyketide synthase
NRPS: non-ribosomal peptide synthetase
FDH: flavin adenine dinucleotide (FADH2)-dependent halogenase
Trp-halogenase: tryptophan halogenase
HPLC-TOF MS: high-performance liquid chromatography time-of-flight mass spectrometry
