# Supplementary material for: Bioprospecting potential of halogenases from Arctic marine actinomycetes
Source: BMC Microbiol. 2016 Mar 10;16:34. doi: 10.1186/s12866-016-0662-2 (PMC4785625; doi:10.1186/s12866-016-0662-2)
Supplement: Additional file 2: — HPLC-TOF MS analysis and qRT-PCR. (PDF 302 kb) [file 12866_2016_662_MOESM2_ESM.pdf]

HPLC-TOF MS analysis and qRT-PCR.

Materials and Methods

HPLC-TOF MS analysis

Fresh culture of strain 604F was inoculated into 2 L fermentation medium containing 5% starch, 1% glucose, 2.5% soy flour, 0.01% KH_2PO_4, and 0.5% KNO_3, which were dissolved in Arctic seawater and adjusted to pH7.2. Fermentation was performed in flasks at 25 °C for 5 days with agitation speed 200 rpm. The fermentation broth was evaporated at 40 °C to dryness under vacuum, and the residue was extracted by methanol (150 mL × 2). The combined extract solution was adjusted to an accurate volume of 300 mL, and then 1 mL of the solution was subjected to analysis on an Agilent 1290 HPLC-6224 TOF MS (Agilent Technologies, Waldbronn, Germany). The HPLC-TOF MS analysis was performed as modified from previous description [1]. In brief, an aliquot of the sample (10 μL) was injected onto a HPLC column (YMC ODS-AA12S03-L546WT, 4.6 × 75 mm, S-5 μm, 12 nm), eluted at a flow rate of 0.5 mL· min^{-1} with a gradient of mobile phase (acetonitrile in water containing 0.1% methanoic acid: 0-1.2 min 5%, 10 min 35%, 15 min 65%, 24 min 95%, 27 min 95%, 27.5 min 5%, 30 min 5%). TOF MS was recorded in positive ion mode ESI in condition of gas temperature, 340 °C; N_2, 8.0 L·min^{-1}; nebulizer, 40 psi. Ion peaks with a mass range of 125 to 1,800, and height over 50,000 were analyzed. Possible formula of each peak was generated by the proprietary molecular formula generator

incorporated into the Agilent MassHunter Workstation software as well as by searching the Dictionary of Natural Products (DNP) database (CRC press), and the calculated exact mass for $[M + H]^+$ was acquired from ChemBio Draw Ultra version 12.0 (CambridgeSoft, Cambridge, MA, USA).

RT-PCR of *hal*604

Total RNA was extracted using Trizol Reagent (Life Technologies) according to the manufacture's protocol, from 1 mL of the above culture of strain 604F. Total RNA was checked by agarose gel electrophoresis and then reverse transcribed into cDNA, which was used as template for PCR amplification of *hal*604 with primers Halo-B4-FW/Halo-B7-RV. The PCR mixture contained 5 μL 10 × PCR buffer (without Mg^{2+}), 3 μL $MgCl_2$, 4 μL dNTPs, 1 μL each primer, 2 μL cDNA, and 0.25 μL Taq polymerase in a final volume of 50 μL. PCR cycling conditions were as below: 95 °C for 3 min, with 30 cycles of 95 °C for 30 s, 55 °C for 30 s, and 72 °C for 1 min, and a final extension at 72 °C for 10 min. PCR products were checked by agarose gel electrophoresis.

Results and Discussion

HPLC-TOF MS analysis and RT-PCR assay

Strain 604F was chosen for primary investigation of natural products using

HPLC-TOF MS, due to the reasons discussed above. The main aim was to identify whether halometabolites were produced by this strain and whether the products were consistent with the prediction. A total of 84 ion peaks were observed in the fermentation broth of strain 604F, which were further analyzed for prediction of possible formulae (Table 1). Up to 61 formulae were given by searching the DNP database, while the remaining 23 observed peaks had no matches to the database. It revealed that the strain 604F produced abundant and diverse natural products under the current conditions. Only one ion peak at *m*/*z* 441.18668 with possible formula of $C_{22}H_{33}BrO_4$ was predicted to contain halogen (Table 1). The formula matched deoxyparguerene in the DNP database. However, upon closer inspection of the MS spectrum, there was no distinct isotopic pattern of bromine (Fig. 1). Therefore, the predicted presence of Br in this formula was probably not reliable. In addition, the RT-PCR amplification of *hal*604 led to no product. Both data suggested that *hal*604 was most likely silent under the current condition. It is a very common phenomenon that secondary metabolite-encoding biosynthetic genes tend to be silent in most cases [2]. Therefore, only a part of natural products were detected in regular isolation and identification. The missing part may indicate great potential of new natural products that can be mined using other improved methodologies. Indeed, various strategies were proposed to explore the cryptic gene clusters, such as one strain/many compounds (OSMAC) strategy, genome sequencing and post-genomic strategies [3]. The halogenase gene-guided bioprospecting is an alternative way to find potential natural products with halogen modification, even when the gene clusters are silent. To

further investigate the putative halogenase gene cluster and the halometabolites of strain 604F, studies such as genome sequencing and optimization of fermentation and LC-MS analysis are underway.

References

1. Wang C, Tian X, Yang Q, Lu Y, Ma L, et al. (2014) Diversity of secondary metabolites from two Antarctic microbes Rhodococcus sp. NJ-008 and Pseudomonas sp. NJ-011. OJMS 04: 214–220. doi:10.4236/ojms.2014.43020.

2. Jensen PR, Chavarria KL, Fenical W, Moore BS, Ziemert N (2014) Challenges and triumphs to genomics-based natural product discovery. J Ind Microbiol Biotechnol 41: 203–209. doi:10.1007/s10295-013-1353-8.

3. Rebets Y, Brötz E, Tokovenko B, Luzhetskyy A (2013) Actinomycetes biosynthetic potential: how to bridge in silico and in vivo? J Ind Microbiol Biotechnol 41: 387–402. doi:10.1007/s10295-013-1352-9.

Table 1. HPLC-TOF MS data of culture broth of strain 604F.

Cpd	Possible formula	Calculated for $[M + H]^+$ (*m/z*)	Base peak (*m*/*z*)	RT (min)	Vol %
**1**	$C_{17}H_{16}O_{10}$	381.0817	381.07946	2.198	3.56
**2**	$C_5H_8O_2S$	150.0583[a]	150.05843	3.236	0.22
**3**	$C_6H_{13}NO_2$	132.1019	132.10216	3.34	1.87
**4**	$C_6H_{13}NO_2$	132.1019	132.10341	3.742	4.39
**5**	$C_9H_8O_3$	182.0811[a]	182.08106	3.784	1.77
**6**	$C_{11}H_{19}NO_8$	294.1184	294.11824	3.988	0.36
**7**	$C_{12}H_{18}N_4O_2$	251.1503	251.15012	4.171	0.63
**8**	$C_{10}H_{13}N_5O_5$	284.0990	284.09908	4.317	0.75
**9**	$C_{10}H_{20}O_4S_2$	269.0876	269.08768	4.351	0.22
**10**	$C_{12}H_{23}NO_7$	294.1548	294.15444	4.415	1.15

**11**			456.20792	4.588	1.34
**12**	$C_{10}H_{19}NO_3$	202.1438	202.1437	4.864	0.87
**13**	$C_{15}H_{27}N_5O_5$	358.2085	358.20787	4.961	2.07
**14**	C_9H_6O	131.0492	131.04884	5.109	0.38
**15**	$C_{10}H_9N^+$	144.0808 [a]	161.10713	5.112	2.57
**16**	$C_{11}H_{14}N_2O_2$	207.1128	207.11261	5.114	2.25
**17**	$C_9H_{11}NO_2$	166.0863	166.08612	5.118	3.34
**18**	$C_{22}H_{33}BrO_4$	441.1835	441.18668	5.143	0.34
**19**	$C_{11}H_{17}NO_7$	276.1078	276.10752	5.172	0.39
**20**	$C_{10}H_{19}NO_3$	202.1438	202.14332	5.248	0.75
**21**	$C_{30}H_{49}O_9P$	585.3187	585.31782	5.293	3.31
**22**	$C_9H_{17}NO_5$	220.1180	220.11786	5.548	1.14
**23**	$C_{25}H_{45}FeN_6O_8$	614.2721	614.27209	5.58	1.39
**24**	$C_{15}H_{22}N_2O_4$	295.1653	295.16512	6.457	0.32
**25**	$C_{13}H_{12}N_2O_2$	229.0972 [a]	246.12357	6.629	1.87
**26**	$C_{11}H_7NO$	170.0601 [a]	187.08632	6.631	0.19
**27**	$C_{11}H_9NO_2$	188.0706	188.07057	6.631	4.07
**28**	$C_{13}H_{17}NO_3$	236.1281	236.12802	6.717	0.67
**29**	$C_{11}H_{20}N_2O_5$	261.1445	261.14433	6.74	0.56
**30**	$C_{14}H_{14}N_2O_2$	243.1128 [a]	260.13931	7.457	0.51
**31**	$C_{12}H_{11}NO_2$	202.0863	202.08611	7.457	1.81
**32**	$C_{36}H_{67}N_{11}O_8S_2$	423.7381 [b]	423.73917	8.422	0.5
**33**	$C_{30}H_{55}N_5O_5$	566.4276	566.42729	9.638	0.71
**34**	$C_{22}H_{20}O_{10}$	445.1129	445.1128	9.656	0.34
**35**	$C_{40}H_{70}O_8$	679.5144	679.51225	10.143	2.48
**36**	$C_{59}H_{96}O_{29}$	1269.6110	1269.61114	10.46	0.15
**37**			792.59546	10.492	2.45
**38**	$C_{37}H_{48}O_4$	557.3626	557.36574	10.514	0.23
**39**			453.34285	10.772	0.99
**40**			218.21124	10.776	0.44
**41**			509.88487	10.995	0.2
**42**	$C_{45}H_{73}NO_{15}$	868.5053	868.50555	11.269	0.68
**43**	$C_{45}H_{73}NO_{14}$	852.5104	852.5104	11.341	0.95
**44**	$C_{13}H_{20}O_3$	225.1485	225.14814	11.406	0.93
**45**	$C_{39}H_{63}NO_{10}$	706.4525	706.45258	11.536	0.51
**46**	$C_{40}H_{65}NO_{15}$	800.4427	800.44162	11.717	0.72
**47**	$C_{25}H_{43}NO_{10}$	518.2960	518.29713	11.943	0.3
**48**			246.24251	13.372	0.51
**49**	$C_{15}H_{10}O_4$	255.0652	255.06486	13.636	2.25
**50**	$C_{16}H_{12}O_5$	285.0758	285.07573	13.79	1.05
**51**	$C_{27}H_{43}NO$	398.3418	398.34124	14.048	0.53
**52**			185.16464	14.079	2.11
**53**	$C_8H_{17}NO$	144.1383	144.13817	14.083	3.34
**54**			870.58087	14.199	0.24

**55**			884.59764	14.553	1.21
**56**			884.59525	14.743	0.61
**57**			1074.68114	14.804	0.24
**58**			898.61261	14.861	0.93
**59**			898.61535	15.002	3.16
**60**			898.61183	15.132	1.42
**61**	$C_{16}H_{35}NO_2$	274.2741	274.27384	15.205	2.26
**62**	$C_{18}H_{39}NO_3$	318.3003	318.29997	15.314	0.96
**63**			912.99452	15.393	0.14
**64**			912.90666	15.397	0.28
**65**			912.83136	15.397	0.1
**66**			912.62781	15.403	6.48
**67**			926.64344	15.548	0.12
**68**	$C_{13}H_{20}O_2$	209.1536	209.1534	15.67	0.74
**69**	$C_{15}H_{10}O_5$	271.0601	271.0597	15.705	0.96
**70**	$C_{13}H_{20}O_2$	209.1536	209.15337	16.002	1.08
**71**	$C_{48}H_{78}O_{18}$	943.5261	943.5266	16.127	0.45
**72**	$C_{18}H_{39}NO_2$	302.3054	302.30522	16.905	0.57
**73**	$C_{13}H_{20}O_3$	225.1485	225.14839	16.908	1.3
**74**			456.27208	17.89	0.53
**75**	$C_{28}H_{39}NO_4$	454.2952	454.2927	21.145	0.69
**76**	$C_{29}H_{39}NO_4$	466.2952	466.29285	21.452	0.42
**77**			907.5785	21.685	0.7
**78**	$C_{28}H_{39}NO_4$	454.2952	454.29278	21.69	4.42
**79**	$C_{38}H_{53}N_9O_9$	780.4039	780.40198	24.085	0.49
**80**	$C_{35}H_{61}O_{15}PS$	785.3542	785.35726	24.086	0.62
**81**			808.43298	24.093	0.37
**82**	$C_{38}H_{53}N_9O_9$	780.4039	780.40126	24.32	0.26
**83**			808.43263	24.321	0.31
**84**	$C_{18}H_{33}NO$	280.2635	280.26311	27.276	0.47

[a] $[M+NH_4]^+$

[b] $[M+2H]^{2+}/2$

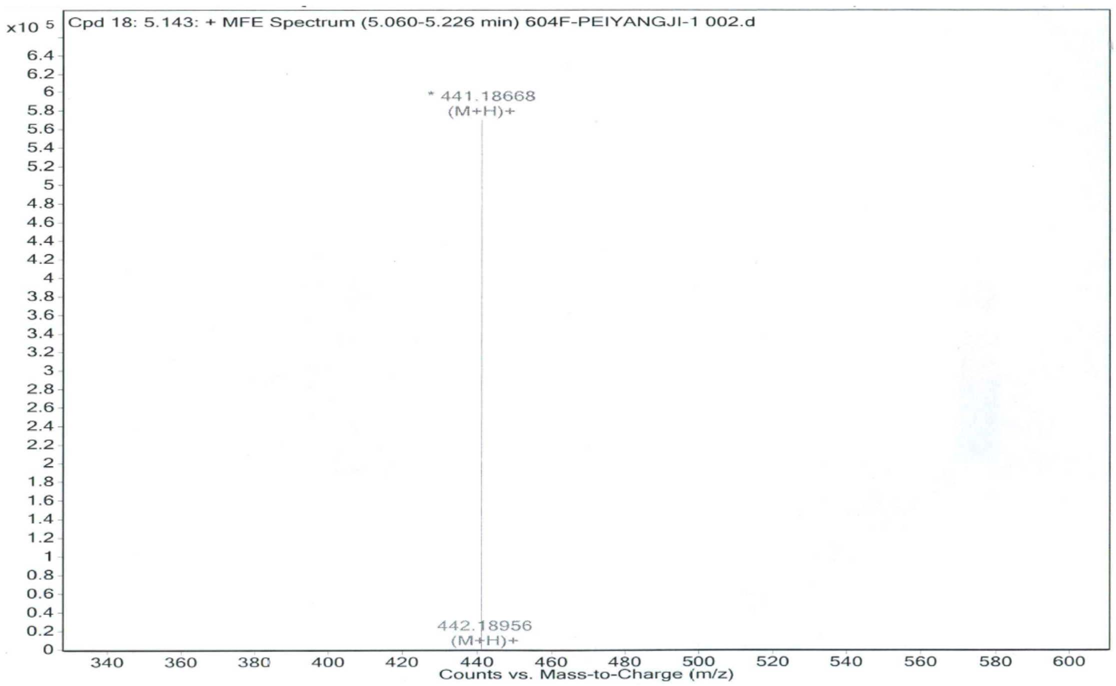

Fig. 1 Extracted positive mode [M + H]+ mass-spectrum for base peak (m/z) 441.18668 at retention time 5.143 min.
